# Development and Validation of an Instrument to Evaluate Perceived Wellbeing Associated with the Ingestion of Water: The Water Ingestion-Related Wellbeing Instrument (WIRWI)

**DOI:** 10.1371/journal.pone.0158567

**Published:** 2016-07-07

**Authors:** Juan Espinosa-Montero, Eric A. Monterrubio-Flores, Marcela Sanchez-Estrada, Inmaculada Buendia-Jimenez, Harris R. Lieberman, François-Andre Allaert, Simon Barquera

**Affiliations:** 1 Nutritional Epidemiology, Nutrition and Health Research Center, Mexican National Institute of Public Health, Cuernavaca, Morelos, México; 2 Academic Operation, Academic Secretary, Mexican National Institute of Public Health, Cuernavaca, Morelos, México; 3 Kidney Health, Danone Research, Palaiseau, France; 4 Independent consultant, Westwood, Massachusetts, United States of America; 5 Medical Evaluation, ESC Dijon-Bourgogne, Dijon, France; Indiana University, UNITED STATES

## Abstract

**Background:**

Ingestion of water has been associated with general wellbeing. When water intake is insufficient, symptoms such as thirst, fatigue and impaired memory result. Currently there are no instruments to assess water consumption associated with wellbeing. The objective of our study was to develop and validate such an instrument in urban, low socioeconomic, adult Mexican population.

**Methods:**

To construct the Water Ingestion-Related Wellbeing Instrument (WIRWI), a qualitative study in which wellbeing related to everyday practices and experiences in water consumption were investigated. To validate the WIRWI a formal, five-process procedure was used. Face and content validation were addressed, consistency was assessed by exploratory and confirmatory psychometric factor analyses, repeatability, reproducibility and concurrent validity were assessed by conducting correlation tests with other measures of wellbeing such as a quality of life instrument, the SF-36, and objective parameters such as urine osmolality, 24-hour urine total volume and others.

**Results:**

The final WIRWI is composed of 17 items assessing physical and mental dimensions. Items were selected based on their content and face validity. Exploratory and confirmatory factor analyses yielded Cronbach's alpha of 0.87 and 0.86, respectively. The final confirmatory factor analysis demonstrated that the model estimates were satisfactory for the constructs. Statistically significant correlations with the SF-36, total liquid consumption and simple water consumption were observed.

**Conclusion:**

The resulting WIRWI is a reliable tool for assessing wellbeing associated with consumption of plain water in Mexican adults and could be useful for similar groups.

## Introduction

The high prevalence of obesity, and the chronic diseases associated with it worldwide, has promoted research to understand the causes, identify treatments and develop strategies to prevent obesity. The contribution of caloric drinks to the development of chronic diseases has been studied in Mexico and their high consumption has been identified as a national problem. Patterns of beverage consumption appear to be based on daily beliefs and knowledge of the population. It has been suggested that increased consumption of plain water may be an effective strategy to decrease development of chronic diseases [1–7].

Research has shown that insufficient fluid intake has various adverse effects on physical and mental performance [8,9]. The use of plain water is recognized as a preferred method for maintaining adequate hydration [10,11]. However, the relationship between ingestion of plain water, hydration and subjective wellbeing has not been evaluated.

Subjective wellbeing is an important component of health that has been defined by the World Health Organization (WHO), as: *“a complete state of physical*, *mental and social wellbeing and not merely the absence of disease or disability”* [12]. Subjective wellbeing has also been defined as the evaluation humans make regarding satisfaction with their own lives, happiness and affective abilities. Wellbeing consists of three components: *wellbeing* itself, the presence or absence of emotions such as enthusiasm, happiness, vitality and love; *discomfort*, which consists of emotions such as sadness, anxiety, lack of enthusiasm and anger, and *satisfaction with life*, which refers to the global assessment an individual makes of current aspects of their daily life such as studies, work, friends, free time, family and health [13–15]. Several investigations have determined that a substantial part of an individual’s daily decisions and behaviors are intended to increase their wellbeing [16–18].

Hydration is an important factor that affects an individual’s perception of their wellbeing. Optimal fluid balance and hydration results from a balance of water loss and water intake. When fluid intake is low or there are losses due to sweating, diarrhea or other causes, total body water decreases and fluid balance is disrupted, activating the sensation of thirst [19]. Depending on the severity of the imbalance, this leads to different levels of discomfort by the individual [20–22]. Mild dehydration (between 1 and 2% body water loss) is associated with negative sensations at the physiological, emotional and cognitive levels, as has been demonstrated in experimental trials with young adults where dehydration was induced with exercise and/or diuretics [8,9]. Physiologically there are sensations of thirst and oliguria [23,24]. Emotionally there are sensations of fatigue, drowsiness, body heaviness, headache, cramps, nausea, lack of enthusiasm and generalized malaise [25,26]. At the cognitive level there are difficulties in concentration, decreased short- and long-term memory, a decrease in motor coordination and slower reflexes [23,25,27,28]. Other sensations that could be related to mild dehidration such as dry skin and constipation have not been completely demonstrated, but have been described in the clinical setting.

Although hydration can alter subjective wellbeing and there are various tools available for evaluating wellbeing [16–18], they were not developed for studying the relationship between wellbeing and hydration. Therefore, the objective of the present study was to develop a tool to evaluate the association of wellbeing with fluid intake–the Water Ingestion- Related Wellbeing Instrument (WIRWI).

## Methods

### Perceptions of wellbeing related to water ingestion

Initially a qualitative study was conducted in 2010 in Cuernavaca Mexico using semi-structured and focus group interview techniques to identify concepts related to wellbeing. Using a theoretical approach based on the phenomenology of wellbeing, we assessed, in a group of volunteers (n = 119), perception of wellbeing in general and the physical and mental dimensions associated with hydration [29].

### Population of the Initial study

The study population consisted of adult males and females who were sedentary or engaged in moderate physical activity with a body mass index (BMI) >18.5. They were between 21–59 years old, lived in Cuernavaca, Mexico and were of low socioeconomic status. The fluid intake was evaluated by 24-hour recall method considering the day before the interview. The selected volunteers were from two quartile-based categories of fluid ingestion: the lowest and the highest quartile. The first quartile consisted of low drinkers with a total fluid intake of <1.5 L and plain water intake of <370 mL. The fourth quartile were individuals who consumed high quantities of fluids, their total fluid intake was ≥2L and plain water intake was ≥1 L. Women who were pregnant or breastfeeding, persons with diseases associated with fluid intake (diarrhea, renal damage, cardiovascular diseases, diabetes or urinary tract infections), or high consumption of alcoholic beverages were excluded from the study [30]. Individuals who had participated in a weight reduction program in the past 6 months were also excluded, as well as those taking any type of multivitamins to control confusion since intake is associated with a positive wellbeing perception, everyday mood and perceived stress [31–33]. **Table 1**shows the distribution and profile of the study population. Recruitment was conducted using radio and local newspaper announcements as well as placement of posters and leaflets in popular, public locations in the city of Cuernavaca. Physical activity was evaluated using the short version of the International Physical Activity Questionnaire (IPAQ) [34]. Socioeconomic level was evaluated using the NSE 10X6, a tool developed by the Mexican Association of Marketing Research and Public Opinion Agencies, A.C. [35]. Body Mass Index (BMI) was defined as kilograms/meters squared (kg/m^2^), WHO classification procedures were used to estimate nutritional status [36]. A digital scale with a variation of 100 g (Model 813, SECA) was used for weight measurement. For measuring height, a portable stadiometer with a 1-mm variation was used (Model 213, SECA). Both measurements were done by trained personnel [37]. The project and its procedures were reviewed and approved by the Commission on Ethics, Research and Biosafety of the National Institute of Public Health in Mexico (Project 896-G67).

**Table 1:** Distribution of semi-structured interviews and focal groups according to profiles in process of development of the WIRWI. Study conducted in 2010 in Cuernavaca, Mexico.

*Technique*	*Groups*	*Sex*	*Body Mass Index*	*n*
***Interviews***	*Light drinkers*	*Male*	*18*.*5–24*.*9*	*8*
		*Male*	*>24*.*9*	*8*
		*Female*	*18*.*5–24*.*9*	*8*
		*Female*	*>24*.*9*	*8*
	*Heavy drinkers*	*Male*	*18*.*5–24*.*9*	*8*
		*Male*	*>24*.*9*	*8*
		*Female*	*18*.*5–24*.*9*	*8*
		*Female*	*>24*.*9*	*8*
***Focal groups***	*Light drinkers*	*Male*	*18*.*5–24*.*9*	*1*
		*Male*	*>24*.*9*	*1*
		*Female*	*18*.*5–24*.*9*	*1*
		*Female*	*>24*.*9*	*1*
	*Heavy drinkers*	*Male*	*18*.*5–24*.*9*	*1*
		*Male*	*>24*.*9*	*1*
		*Female*	*18*.*5–24*.*9*	*1*
		*Female*	*>24*.*9*	*1*

### Development of the WIRWI

Based on the initial qualitative study we developed guides for focus groups and for semi-structured interviews to explore subjective wellbeing associated with consumption of plain water. Focus groups and interviews conducted by experienced field researchers using standardized procedures. Interviews were audio-recorded after verbal informed consent was obtained and were conducted in locations designated by the participants. Sixty-four interviews and eight focus groups were conducted for each demographic group as defined in Table 1 (eight interviews and one focus group for each sub-group). Recordings of the interviews and focus groups were transcribed by trained personnel based on a standardized guide developed using Atlas Ti, a specialized software program [38]. Analysis files were created for the following categories of daily life: perception of wellbeing, knowledge, attitudes, beliefs, and other perceptions. Data analysis consisted of consolidating the testimony of participants and independently comparing the data collected during the focus groups and interviews [39]. The analysis consisted of constructing an inventory of the volunteers’ observations regarding wellbeing and health aspects associated with hydration. Construction of individual items was based on the criteria of Moriyama [40]. Based on the findings of this stage, the final design of the WIRWI was determined with assistance of a scientific advisory board with expertise in hydration and psychometric analysis.

### Validation

The process of validation consisted of five stages. First, after analyzing the 146 items obtained from the qualitative study they were tested quantitatively and qualitatively in a pilot study to evaluate understanding and acceptance. The expert group designed a questionnaire that grouped similar items and asked which item in each group was simpler to understand. This survey was then applied to 50 volunteers [25 women (average age 34.7 ± 9.5y) and 25 men (average age 36.5 ± 11.1y), from low socio-economic status]. From this volunteers, 16 participants were included in two focus groups to discuss comprehension of each item. Using this information, adjustments were made to some items and those with better understanding scores were selected. Finally, using the Rash methodology, all items were evaluated based on the theory of responses to an item [41]. Fifty-five items were selected based on this pilot study and expert opinion **S1 Table**. In the second stage, content validation and face validation were assessed.

This fifty-five item instrument was reviewed by the outside experts on development and validation of psychometric instruments who evaluated and classified the items based on their physical and mental dimensions. The experts ruled on the relevance of each item and offered recommendations. The study population for subsequent stages consisted of adults with the same characteristics as participants in the qualitative study. In the third stage, an exploratory factor analysis was conducted with data from 682 participants. Variables with factor loadings ≥0.4 or with high contrast between dimensions were evaluated and different rotations were tested. A reliability analysis was done for the general model and for each dimension identified, with Cronbach’s alpha set at ≥0.80. In this stage, we evaluated the contribution of each item to the wellbeing construct eliminating those with poor contribution or those explaining other dimensions. In the fourth stage, a confirmatory factor analysis was done with the data from 415 participants using the statistical criteria of Boomsma (31) and McDonald and Ho [42,43] to select the best model. In the fifth stage, data from 315 persons (173 not recruited and 142 recruited), who participated in an eight week long clinical trial of a water consumption intervention designed to assess perception of wellbeing associated with hydration were analyzed (Clinical Trial NCT01982981, US National Institutes of Health). In this intervention, participants who were not recruited were evaluated on two occasions, recruitment stage (one week before to baseline) and baseline. Participants who were recruited were evaluated on four occasions: recruitment stage, baseline, the second week and the eighth week after baseline. During this stage, internal validity, reproducibility, concurrent validity and sensitivity to change of the proposed WIRWI items were evaluated.

**Fig 1:**
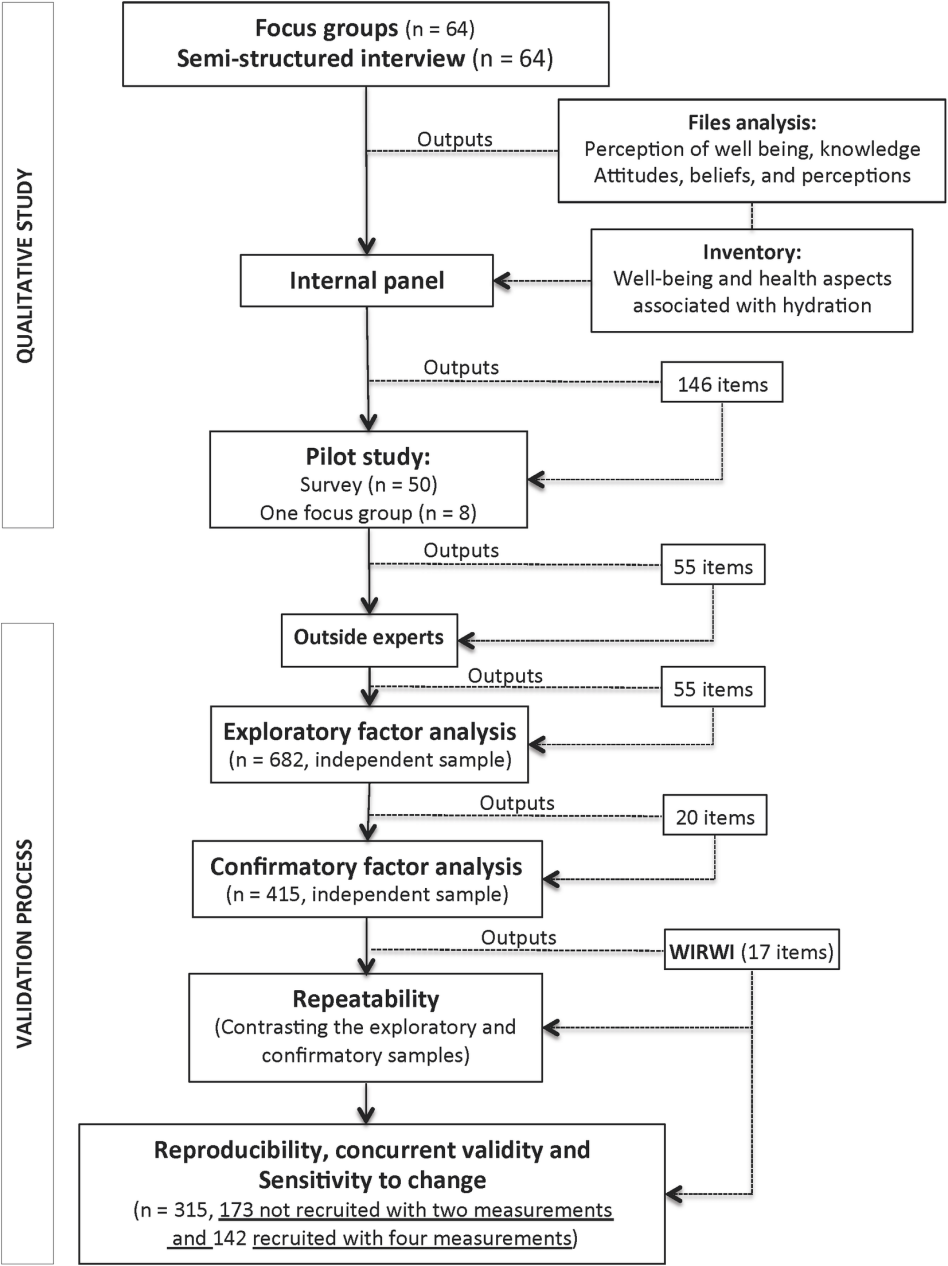
Process of development and validation of the Water Ingestion- Related Well Being Instrument in adult Mexican. Study conducted in 2010 in Cuernavaca, Mexico.

The internal validity was evaluated contrasting the Cronbach’s alpha from the responses in the exploratory and confirmatory factorial analysis. The reproducibility was evaluated with the Spearman correlation of total WIRWI score, physical and mental scores between two measures to every one person, intra class coefficient (ICC) and the tau kendall correlation to every item. Concurrent validity was evaluated by correlation of the global score and using the following measures: 1) the dimension of Physical Health Composite Score (PCS) and Mental Health Composite Score (MCS) as assessed by the SF-36 instrument [44–46], 2) 24-hour Urine Osmolality (UO) and 24-hour urine total volume 3) total consumption of fluids from beverage intake and plain water (fluids from foods were not considered). In addition, concurrent validity was evaluated comparing the mean differences of the WIRWI score by osmolality tertiles. Sensitivity to change was evaluated by measuring the effect of the intervention on perception of wellbeing, compared to changes observed using the SF-36. Statistical analysis was conducted using the software packages Stata v.12.0 and EQS v.6.1 [47]. Urine was collected using standardized procedures [48]. Trained personnel determined UO with a micro-osmometer using the freezing point technique (Fiske Micro-osmometer, Model 210; Advanced Instruments, Inc). The data analysis was carried out in 2013. **Fig 1**presents the different procedures conducted to develop the WIRWI.

## Results

From the qualitative study we obtained a total of 146 items and from those 55 items were selected in the pilot test. Based on the Rasch methodology we found that infit and outfit for all items were in an acceptable range (0.95 a 1.05). These items were classified according to two dimensions: the physical dimension (19 items) and the mental dimension (36 items). The instrument assessed perceived wellbeing during the prior week and used a Likert-type response scale with five options: *always*, *almost always*, *sometimes*, *almost never* and *never*.

The exploratory factor analysis using varimax rotation and forcing two factors with values of 10 and 4, respectively, explained 32% of the variance (Keiser-Meyer-Olkin test was 0.90). Only those variables with loadings ≥0.45 or contrasting values between factors and with theoretical congruence were considered. The final model included 20 items for the physical (n = 10) and mental (n = 10) dimensions with Cronbach’s alpha = 0.87 for the overall model and a reliability coefficient of 0.97 (physical dimension Cronbach’s alpha = 0.82, mental dimension Cronbach’s alpha = 0.86). Selected items along with their factor loadings are shown in **Table 2**.

**Table 2:** Final structure of the exploratory analysis of the Water Ingestion-Related Wellbeing Instrument with two components in process of validation of the WIRWI. Study conducted in 2010 in Cuernavaca, Mexico.

Item numberDescription of the item		Mental Dimensionfactor loading	Physical Dimensionfactor loading
1	My skin feels dry	-0.0695	**0.6351**
2	My lips are parched	-0.089	**0.5973**
3	My nails are brittle	-0.1615	**0.5616**
4	My hair is dry	-0.1581	**0.6326**
6	I have bad breath	-0.1216	**0.4139**
9	I feel stress or shortness of breath when climbing stairs	-0.2217	**0.4744**
11	I have cramps or muscle aches	-0.1498	**0.5675**
15	I have headaches	-0.1497	**0.5154**
16	I am constipated	-0.1405	**0.5436**
17	I have stomach discomfort (pain, bloating, gas)	-0.1545	**0.5806**
26	I am alert and react quickly to what is happening around me	**0.4183**	0.096
27	I have good concentration for my activities	**0.6421**	-0.0481
29	I successfully completed tasks requiring concentration and reflection	**0.7293**	0.0062
31	I am active in carrying out my daily activities	**0.6488**	-0.0463
33	I have a positive interest in carrying out my daily work	**0.6059**	-0.1246
36	I feel that my memory is good	**0.5119**	-0.1278
43	I am happy or animated	**0.6278**	-0.3232
44	I feel optimistic about life and my activities	**0.6066**	-0.2397
47	I feel confident and self-assured	**0.6301**	-0.1255
50	I feel confident in decision-making	**0.5767**	-0.15

**Fig 2:**
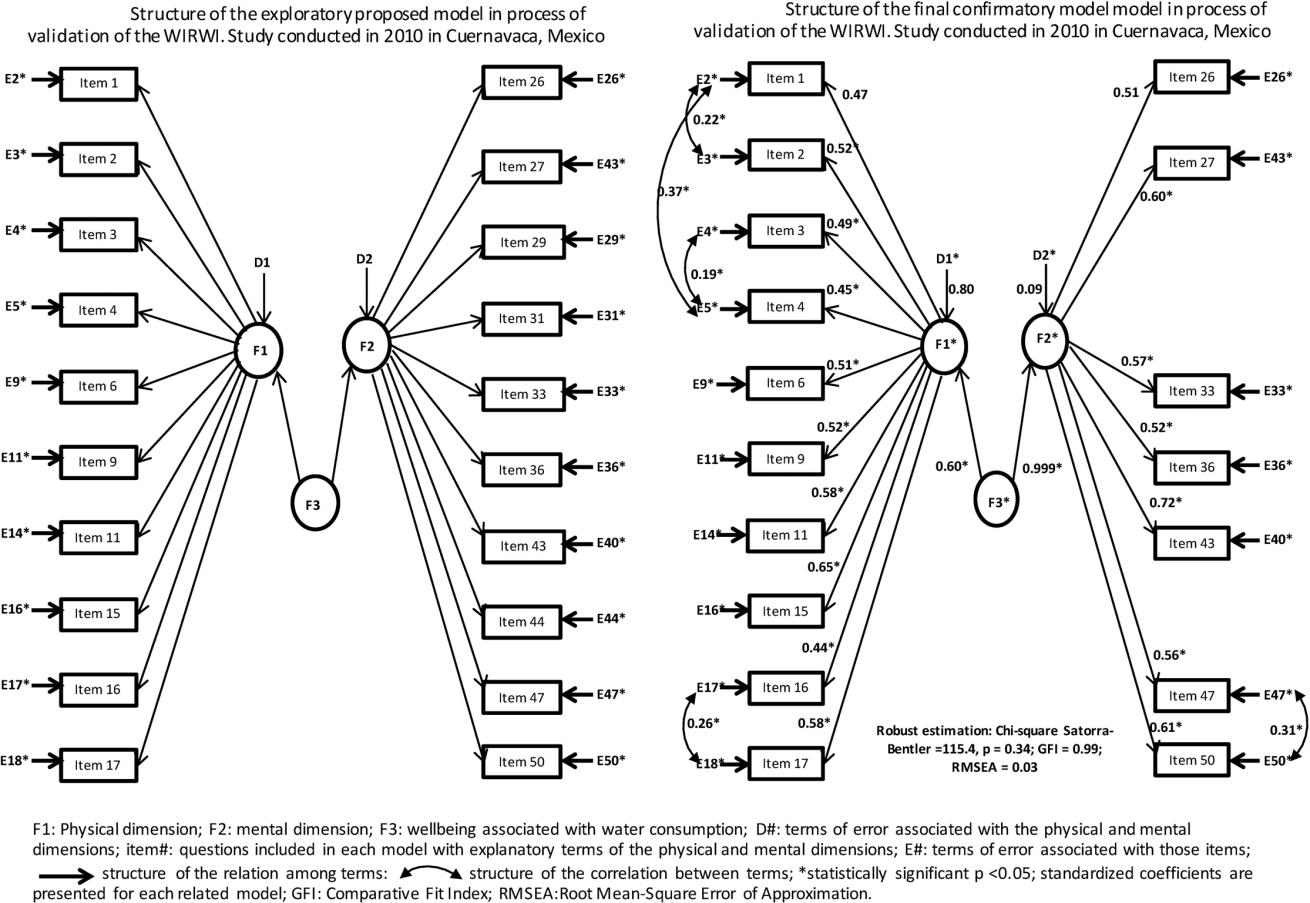
Structure of the exploratory proposed model in process of validation of the WIRWI and Structure of the final confirmatory model model in process of validation of the WIRWI.

For the confirmatory factor analysis, the empirical structure obtained in the exploratory analysis was used *a priori* (**Fig 2**). Ten proposed items were retained for the physical (Cronbach’s alpha = 0.81) and seven items for the mental (Cronbach’s alpha = 0.81) dimensions. Items 29, 31 and 44 were excluded using as elimination criteria non-significant values or when Lagrange multipliers indicated a relationship different from that established in the proposed construct. Cronbach’s alpha for the general model was 0.86. The final model fit was evaluated using the following estimations: Satorra-Bentler test (χ^2^ = 115.4, *p* = 0.344), Bentler-Bonett Normed Fit Index (0.925); Bentler-Bonett Nonnormed Fit Index (0.995), Comparative Fit Index (0.996), McDonald Fit Index (0.996) and Root Mean-Square Error of Approximation (0.011, 90% CI 0.01, 0.28) (**Fig 2**). The final version of WIRWI consists of 17 items **Table 3**(WIRWI in original Spanish language is shown in **S2 Table**).

**Table 3:** The final version of *Water Ingestion-Related Wellbeing Instrument* consists of 17 items. Study conducted in 2010 in Cuernavaca, Mexico.

Item number	Description of the item
1	My skin feels dry
2	My lips are parched
3	My nails are brittle
4	My hair is dry
6	I have bad breath
9	I feel stress or shortness of breath when climbing stairs
11	I have cramps or muscle aches
15	I have headaches
16	I am constipated
17	I have stomach discomfort (pain, bloating, gas)
26	I am alert and react quickly to what is happening around me
27	I have good concentration for my activities
33	I have a positive interest in carrying out my daily work
36	I feel that my memory is good
43	I am happy or animated
47	I feel confident and self-assured
50	I feel confident in decision-making

*Metric Proposed for the WIRWI*. All items in the WIRWI had the same relationship to wellbeing, where 0 = poor wellbeing and 4 = optimal wellbeing. The highest possible score is 68 points and the lowest is 0. The raw score obtained is transformed into a percent where the highest value (68 points) equals 100% and the lowest value is 0%. This score is calculated using the following equation:

$$\text{Wellbeing Index}\;(\%)\;=\frac{100}{68}\;\sum_{i=1}^{50}\;{ítem}_{i}$$

Where "i" = 1, 2, 3, 4, 6, 9, 11, 15, 16, 17, 26, 27, 33, 36, 43, 47, 50 item

For the sample obtained in the exploratory factorial analysis and in the verification process the wellbeing index was correlated with each item to ensure cohesiveness with the index. In all cases, the association was positive (correlations between 0.25 and 0.43, p <0.001). Kendall’s tau was used as test statistic.

The repeatability was acceptable, the Cronbach’s alpha of the global model and from the physical dimension were similar (Feldt test, p > 0.19) in both samples (exploratory and confirmatory independent samples); in the mental dimension the Cronbach’s alpha was different (Feldt test, p < 0.001).

Acceptable reproducibility was also observed; **Table 4**presents the tests on differences in Cronbach’s alpha between recruitment stage and baseline measurement on the same sample of 315 participants (173 not recruited and 142 recruited). The Spearman correlation between recruitment stage and baseline from the WIRWI score were 0.77, 0.71 and 0.72 for the global model, physical and mental dimensions respectively (statistically significant in all cases, p < 0.0001). The interclass coefficients were 0.76, 0.69 and 0.71 for de global model, physical and mental dimensions respectively. The correlations for each item varied from 0.43 to 0.61 (statistically significant in all cases, p < 0.0001).

**Table 4:** Internal consistency and the test of the global model and by physical and mental dimensions in a sample* with two measurements. Study conducted in 2010 in Cuernavaca, Mexico.

Measurements	Global model		Physical dimension		Mental dimension	
	Cronbach’s Alpha	Feldt test p	Cronbach’s Alpha	Feldt test p	Cronbach’s Alpha	Feldt test p
Recruitment stage	0.86	0.94	0.82	0.83	0.81	0.83
Baseline	0.89		0.84		0.87	

*n = 315 (173 not recruited and 142 recruited)

In the concurrent validation process correlations of the WIRWI with the different components of the SF-36 (vitality, physical and mental) at the tree measurements (baseline, second and eighth week) were statistically significant (p <0.001 for all cases); the correlations with vitality had a range from 0.62–0.72; physical from 0.31–0.52 and mental from 0.59–0.74. The WIRWI correlations to total fluid consumption at baseline, second and eighth week measurements were respectively 0.21 (p = 0.010), 0.32 (p <0.001) and 0.17 (p = 0.048). The correlations with water consumption were statistically significant at the baseline and eighth week measurements, 0.19 (p = 0.025) and 0.2 (p = 0.020) respectively. No statistically significant correlations were observed between the instrument and UO and 24-hour urine total volume at each measurement period; however, when the three measurements were combined, a correlation of 0.11 (p = 0.044) was observed.

A significant difference was observed between average WIRWI by UO tertile (p ≤0.001). The first tertile (UO = 331.5; 95% CI = 67, 515) had the highest average WIRWI, the second tertile (UO = 652.0; 95% CI = 522, 763) was 4 points lower and the third tertile (UO = 901; 95% CI = 764, 1191) was 5.8 units lower relative to the first tertile. However, the trend-test was not significant (p = 0.793).

For the sensitivity to change, the effect of the intervention evaluated using the WIRWI score was 3.3 percent points (pp) at second week (p = 0.03) and 6.5 pp at the eighth week (p = 0.001). In the physical dimension, the score was 3.7 pp in the second week (p = 0.069) and 8.1 pp in the eighth week (p< 0.001). In the mental dimension, the score was 3.7 pp in the first week (p = 0.07*) and 4.7 pp in the third week (p = 0.027). The WIRWI score in the control group increased 2.2 percent points (pp) at second week (p = 0.01) and 3.5 pp at eighth week (p = 0.002). In the intervention group increased 5.6 pp at second week (p <0.001) and 9.9 pp at eighth week (p <0.001). Similar effects to the WIRWI were observed using the SF-36 instrument; the effect of the intervention evaluated using the SF-36 component score was, in PCS 2.0 pp in the eighth week (p = 0.019), and MCS 3.7 pp in the second week (p = 0.002) and 5.2 pp in the eighth week (p < 0.001).

## Discussion

The present study was the first, to the best of our knowledge, to develop and validated the instrument designed to evaluate wellbeing associated with water ingestion. These procedures used were based on development of an in-depth understanding of the topic. Unlike similar instruments, the WIRWI was developed using all the recommended stages for construction and validation of such questionnaires [40,49,50]. The process included validation of the content, construct and criteria and tests for measuring reliability, reproducibility and sensitivity to change. Procedures for measuring reliability and comparison to physiological indicators and fluid ingestion were also employed. The instrument is also correlated with components of a health and quality of life questionnaire and with total intake of fluids (including water), which improves its validity. We also demonstrated the WIRWI was sensitive to changes in subjective wellbeing associated with variations in water ingestion (concurrent validity). The instrument was not correlated with 24-hour urine total volume or UO. This lack of correlation has been observed in other studies when instruments based on perception have been compared to biochemical parameters. This may be due to the fact that the measurements of UO and 24-hour urine total volume were conducted on the day prior to the interview, while the WIRWI assessed perception over the previous seven days. The instrument also evaluates attitudes associated with physiological processes related to hydration but does not consider other factors that could influence perception of wellbeing such as beliefs, attitudes, and feelings that people have with respect to their health and are part of their individual experience [51]. Despite the fact that evaluation of perceived wellbeing is considered to be subjective, it has been associated with objective indicators of health such as the presence of chronic diseases and their severity, predictors of disease, longevity and increased duration of hospitalization [51–53].

Our instrument was developed using empirical data from a low-income adult population in an urban area of a Mexico. In our view this approach is important because this group is very large in many countries, including Mexico, where non-communicable chronic diseases are rapidly increasing and understanding water ingestion-related wellbeing might be of great benefit [54–56]. To build the WIRWI, we focused on participant’s perceptions of wellbeing related to hydration. Other instruments such as the Guttman Scale for Water Insecurity have been developed using the same approach to evaluate the effect on psychological stress and mental health [57–61]. Since the WIRWI was constructed independently from the water insecurity context, it could be an additional useful tool to explore wellbeing in populations with this condition.

Nevertheless, caution should be exercised when considering the use of the WIRWI in other populations with different socio-economic and cultural conditions. For example, item 9 refers to the physical condition after climbing stairs. In some poor populations where stairs are not common, this wording could not be effective and an adequate adaptation of the instrument might be necessary.

The prevalence of many chronic illnesses, such as diabetes and kidney disease, reported to be associated with consumption of sugar-sweetened beverages is increasing [62–65]. The WIRWI provides a validated method that quantifies perceived wellbeing associated with water intake, and therefore can be used to understand from a quantitative approach, the perception of wellbeing in individuals might choose to hydrate with sugar-sweetened beverages rather than plain water). During our review we did not find other instruments designed to evaluate wellbeing associated with hydration, thus direct comparisons were not possible. We believe our instrument could readily be adapted to other populations to evaluate: 1) wellbeing associated with water ingestion and hydration; 2) physical activity; 3) programs designed to promote greater fluid consumption; and 4) beliefs and perceptions in health and disease. The WIRWI could also be used in vulnerable groups where adequate hydration is of great relevance, such as children and elderly people [66].

## Supporting Information

S1 Table Items grouped into dimensions for validation by judges. (DOCX)

S2 Table The final version of Water Ingestion-Related Wellbeing Instrument in original Spanish language. (DOCX)
