# Supplementary material for: Development and Validation of an Instrument to Evaluate Perceived Wellbeing Associated with the Ingestion of Water: The Water Ingestion-Related Wellbeing Instrument (WIRWI)
Source: PLoS One. 2016 Jul 7;11(7):e0158567. doi: 10.1371/journal.pone.0158567 (PMC4936734; doi:10.1371/journal.pone.0158567)
Supplement: S1 Table — (DOCX) [file pone.0158567.s001.docx]

**S1 Table.** **Items grouped into dimensions for validation by judges in process of development WIRWI. Study conducted in 2010 in Cuernavaca, Mexico.**

| **Item**  **number** | **Item description** |
| --- | --- |
|  | *Physical dimension* |
| 1 | My skin is dry |
| 2 | My lips are dry |
| 3 | My nails are brittle |
| 4 | My hair is dry |
| 5 | I feel very thin |
| 6 | I have bad breath |
| 7 | My clothes feel tight |
| 8 | When climbing stairs I feel agitated or short of breath |
| 9 | My physical condition is better in performing my daily activities |
| 10 | I have cramps or muscle aches |
| 11 | I run, walk or go to the gym to stay in shape |
| 12 | My throat is dry |
| 13 | My urine is dark |
| 14 | I have headaches |
| 15 | I am constipated |
| 16 | I have stomach distress (pain, inflammation, gas) |
| 17 | I am thirsty |
| 18 | I feel hungry between meals |
| 19 | I try to eat healthier |
|  | *Mental dimension* |
| 20 | I feel tired when walking, climbing stairs, doing housework and, in general, any daily activities |
| 21 | I have more desire to live with others |
| 22 | I get along well with persons nearby without any fights or arguments |
| 23 | I feel comfortable with my friends and neighbors |
| 24 | I am bothered by people in the street asking me questions |
| 25 | I am intolerant of people with whom I interact |
| 26 | I am alert and react quickly to my surroundings |
| 27 | I have good concentration for my activities |
| 28 | Time goes by quickly because I am dedicated to my activiites |
| 29 | I feel that I successfully complete my tasks that require concentration and reflection |
| 30 | I feel absent-minded |
| 31 | I am active when carrying out my daily activities |
| 32 | I have little energy when carrying out my daily activities (work, study, sports, chores) |
| 33 | I feel like carrying out my daily work |
| 34 | I feel listless for carrying out any activity |
| 35 | I feel rested upon awakening in the morning |
| 36 | I feel that my memory is good |
| 37 | I easily forget things |
| 38 | I effortlessly remember any data or information |
| 39 | I remember important dates |
| 40 | I feel happy in developing my activities |
| 41 | I have positive thoughts |
| 42 | I feel desperate in everyday activities (waiting for a bus, waiting in line to make a purchase or paying for a service along with others) |
| 43 | I feel content or animated |
| 44 | I feel optimistic about life and its activities |
| 45 | I feel stressed |
| 46 | I feel less confident than others |
| 47 | I feel confident and secure |
| 48 | I feel satisfied with my physical appearance |
| 49 | I accept the way I am |
| 50 | I feel confident in decision-making |
| 51 | It is difficult for me to sleep |
| 52 | I have insomnia during the night |
| 53 | I sleep during the day |
| 54 | I awake at various times during the night |
| 55 | I sleep easily |
