# Supplementary material for: Development and Validation of an Instrument to Evaluate Perceived Wellbeing Associated with the Ingestion of Water: The Water Ingestion-Related Wellbeing Instrument (WIRWI)
Source: PLoS One. 2016 Jul 7;11(7):e0158567. doi: 10.1371/journal.pone.0158567 (PMC4936734; doi:10.1371/journal.pone.0158567)
Supplement: S2 Table — (DOCX) [file pone.0158567.s002.docx]

**S2 Table. The final version of *Water Ingestion-Related Wellbeing Instrument* in original Spanish language. Study conducted in 2010 in Cuernavaca, Mexico.**

| **Item number** | **Description of the item** |
| --- | --- |
| 1 | Sentí la piel reseca |
| 2 | Sentí los labios resecos |
| 3 | Noté mis uñas quebradizas |
| 4 | Noté mi cabello reseco |
| 6 | Me percaté de que tuve mal aliento |
| 9 | Al subir escaleras me sentí agitado(a) o con falta de aire. |
| 11 | Tuve calambres o dolores musculares. |
| 15 | Tuve dolores de cabeza. |
| 16 | Tuve estreñimiento. |
| 17 | Tuve molestias estomacales (dolor, inflamación, gases). |
| 26 | Estuve alerta y reaccioné rápidamente a lo que pasaba a mí alrededor. |
| 27 | Me concentré con facilidad para realizar mis actividades. |
| 33 | Me sentí con ganas de hacer mis labores diarias. |
| 36 | Sentí que mi memoria fue buena. |
| 43 | Me sentí contento(a) o animado(a) |
| 47 | Me sentí confiado(a) y seguro(a) |
| 50 | Me sentí seguro(a) para tomar decisiones. |
